# Understanding the behavioural determinants of opioid prescribing among family physicians: a qualitative study

**DOI:** 10.1186/s12875-019-0947-2

**Published:** 2019-05-10

**Authors:** L. Desveaux, M. Saragosa, N. Kithulegoda, N. M. Ivers

**Affiliations:** 1 Women’s College Research Institute, Women’s College Hospital, 76 Grenville Ave, Toronto, ON M5S 1B2 Canada; 2 Institute for Health Policy, Management & Evaluation, University of Toronto, 155 College Street Suite 425, Toronto, ON Canada; 3 Family Practice Health Centre, Women’s College Hospital, 76 Grenville Ave Toronto, Toronto, ON Canada; 4 Institute for Health System Solutions and Virtual Care, Women’s College Hospital, 76 Grenville St, Toronto, ON Canada

**Keywords:** Qualitative, Theoretical domains framework, Prescribing, Opioid

## Abstract

**Background:**

Longstanding variation in the views of family physicians (FPs) on the role of opioids seems to translate into widely varying prescribing rates. Improvement interventions are unlikely to achieve change if they do not understand and explicitly target the factors that determine physician prescribing behaviour. The aim of this work was to understand (1) the perspectives of FPs as it relates to opioid prescribing, and (2) the perceived barriers and enablers to guideline-adherent opioid prescribing and management of chronic non-cancer pain.

**Methods:**

A qualitative study involving one-on-one, semi-structured interviews with a sample of FPs in Ontario, Canada. Interviews were analyzed using a directed content analysis informed by the Theoretical Domains Framework. A framework approach was used to explore interaction across behavioural determinants (factors influencing behaviour) as well as demographic sources of variation. The behaviour of interest for the current study was the prescribing of opioid medications (including initiation, renewal, and dose reduction) for patients with chronic, non-cancer pain. Associated issues in the overall management of such patients were also explored.

**Results:**

Interviews were conducted with 22 FPs. Behavioural determinants interacted with one another to influence FPs prescribing behavior. The TDF domain *Beliefs about Consequences* played a central role in explaining physician prescribing behaviours as they related to the management of chronic non-cancer pain. Individual beliefs about prescribing consequences and patient behaviour interacted with prescriber beliefs about capabilities and perceptions of the FP’s professional role to influence prescriber behaviour. Emotion and the environmental context influenced the impact of these determinants on opioid prescribing and the management of chronic non-cancer pain.

**Conclusions:**

FPs face a wide range of complex (and often interacting) challenges when prescribing opioid therapy to their patients. Solution-based strategies should target these determinants directly using evidence-based strategies that move beyond guideline dissemination and general education. Shared decision-making strategies and patient-facing decision aids are likely to decrease the tension experienced in challenging conversations.

**Electronic supplementary material:**

The online version of this article (10.1186/s12875-019-0947-2) contains supplementary material, which is available to authorized users.

## Background

Opioids are used to treat a broad range of chronic non-cancer (CNCP) pain despite a dearth of evidence regarding safety and efficacy [1–3]. In Canada, the number of opioid prescriptions, and the proportion of ‘strong’ opioids (i.e., oxycodone, hydromorphone, morphine, and fentanyl) increased from 2012 to 2016 (by 6.8 and 5.1%, respectively) [4]. The shift away from low dose opioids (i.e., codeine) towards more potent opioids is a concern in the province of Ontario, where the number of people who filled a prescription for hydromorphone increased by 29% from 2013/14 to 2015/16 [5]. However, an 18% dose reduction was observed (in milligrams or morphine or morphine equivalent) between January 2015–March 2017, driven by lower volumes of dispensed long-acting opioids [6]. This shift coincided with a range of public health initiatives [6], suggesting a level of responsiveness from the medical community to emergent evidence on opioid risks.

Despite this shift, family physicians (FPs) in Ontario demonstrate widely varying prescribing rates. The highest prescribers provide opioid prescriptions 55 times more often than their lowest prescribing colleagues, suggesting that FPs might be able to reduce the risk of opioid-related harm by writing fewer opioid prescriptions [7]. Longstanding variation in the views of FPs on the role of opioids may be a contributing factor, presenting a possible target for behaviour change interventions. In 2001, 35% of Canadian FPs reported they would never use opioids for chronic, non-cancer pain; however 32% described them as the preferred option in this population [8]. Almost 10 years later, variations in opioid-related knowledge and self-reported practice patterns persisted [9].

Knowledge-practice gaps reduce the impact that potentially effective interventions can have on public health [10, 11]. Implementation research aims to bridge this gap by investigating methods to promote evidence uptake by healthcare professionals’ [12, 13]. This includes identifying key factors associated with healthcare professional behavior, which provides an evidence base to inform the development and delivery of initiatives to increase guideline-concordant care [12].

Given the extent of historic variation, there is a need to understand the current determinants of opioid prescribing among FPs to accurately inform solutions that aim to influence behavior in the current prescribing context. These insights will also provide a baseline assessment of the determinants of physician behavior in advance of newly developed initiatives. The aim of this work was to understand (1) the current perspectives of FPs as it relates to opioid prescribing, and (2) the perceived barriers and enablers to guideline-adherent opioid prescribing and management of CNCP.

Canadian guidelines for opioid therapy and the management of CNCP were updated in May 2017, recommending the use of non-pharmacological and non-opioid treatments as a first-line approach to care, tapering opioid doses in individuals already receiving these drugs, and restricting the maximum prescribed dose to 90 mg morphine equivalents daily [14]. The guidelines recommend against the use of opioids among individuals with active substance use disorder and those with active psychiatric disorders whose condition is not yet stable, as well as strongly suggesting that the maximum prescribed dose be restricted to less than 50 mg of morphine equivalent dose when opioid therapy is being initiated.

## Methods

This qualitative study involved a series of one-on-one, semi-structured interviews conducted with a sample of FPs in Ontario, Canada in July and August 2017. This work was conducted within the Ontario Health Implementation Laboratory- a partnership between implementation scientists and Health Quality Ontario (HQO), designed to support the evaluation and refinement of quality improvement initiatives within the Ontario health system. HQO is the provincial advisor on quality standards and performance across the health care system. In this role, HQO distributes Practice Reports to FPs that contain aggregated data on their patients’ cancer screening, diabetes management, and health service utilization, as well as opioid prescribing [15]. HQO also collaborates with partner organizations across the health system on a coordinated program of supports to help clinicians manage their patients’ pain, including the appropriate use of opioids [16]. This project stemmed from a desire to inform those initiatives.

The protocol received ethics approval from the Women’s College Hospital Research Ethics Board.

### Study setting

In Ontario, Canada, primary care is considered the first point of contact between a patient and the health care system and can cover illness prevention, health promotion, diagnosis, treatment, rehabilitation, and counseling [17]. FPs play a role as gatekeepers, whereby they often authorize access to specialty care and diagnostic imaging. Primary care has evolved from primarily a fee-for-service (FFS) model of solo practicing physicians to group-based practices founded on patient enrolment and comprehensive care, with less than a quarter of physicians compensated by a FFS model in 2015 [18]. Approximately 90% of Ontarians report having a regular family physician [19], with over 24% enrolled in primary care practices characterized by the presence of multidisciplinary teams funded by the government in addition to FPs (e.g., community health centres, family health teams) [20, 21]. The rest of Ontarians receive care from FPs who often practice without the presence of allied health care professionals as part of the primary care team.

### Recruitment

Recruitment involved a two-tiered strategy. In partnership with HQO, we invited FPs who had requested to receive a Practice Report (*n* = 924) to participate in a one-time telephone interview on their perceptions of and experiences with prescribing and managing opioid therapy for patients in their practice. FPs received an email invitation outlining details of the study, advising them to contact the study coordinator if they were interested in participating. A follow-up recruitment email was sent within 2 weeks of the original invitation. A total of 10 participants contacted the study coordinator to express interest and all were included in the study.

A purposive sampling strategy was also employed to ensure the results reflected both rural and urban FPs and the range of possible practice and remuneration structures in primary care. A member of the research team sent a recruitment email via an existing network of FPs to mitigate bias and ensure representation from those who were not signed up to receive HQO’s practice report. Of the 12 emails sent, all 12 potential participants expressed interested and contacted the study coordinator to schedule the interview. Participants were provided with the informed consent form via email at least 48 h prior to their interview to enable sufficient time for review. Immediately prior to conducting the interview, participants provided verbal consent that acknowledged their understanding for each component to the study. In response to early data analysis, this recruitment strategy targeted FPs in a fee-for-service remuneration model to seek disconfirming evidence for the emergent themes.

### Data collection

All but one semi-structured interview occurred over telephone and were audio recorded (one interview was conducted in person). Interview questions explored perceptions of opioid prescribing and barriers and facilitators to guideline-based care. Questions that formed the semi-structured interview guide were developed by the research team based on the Theoretical Domains Framework (TDF) (see Additional file 1). The TDF is a validated framework that helps to systematically uncover determinants of individual behaviour [22]. The TDF includes 84 determinants across 14 domains based on psychological theory and has been used extensively to understand behavior in order to inform the design of complex healthcare interventions [23–26]. The behaviour of interest for the current study was the prescribing of opioid medications (including initiation, renewal, and dose reduction) for patients with chronic, non-cancer pain. Associated issues in the overall management of such patients were also explored when they informed the physician’s behaviour.

Demographic details, including sex, years of practice, and practice type, were recorded prior to the interview. Interview recordings were transcribed verbatim and anonymized prior to analysis.

### Data analysis

Interview transcripts were independently coded by two members of the research team (MS and NK) using directed content analysis where individual TDF domains were applied as deductive codes [26]. Coding guidelines were generated to ensure consistency and establish an audit trail of the original data to final themes [26, 27].

Initial themes reflected the TDF domains that emerged as determinants of FP behavior. These themes were defined as those that directly influenced the key behaviour (opioid prescribing) across multiple transcripts [26]. Data were coded to multiple domains where appropriate (referred to as the *intersection* of behavioural determinants). Following the identification of key domains, a framework approach was used to explore demographic sources of variation within the dataset [27, 28], including years of practice, geography, and practice type. Patterns emerged according to individual beliefs (corresponding to the TDF domain *Beliefs about Consequences*), whereby coding for this domain often intersected with coding for other domains. Therefore the authors constructed a matrix to explore how individual beliefs interacted with other behavioural determinants to impact opioid prescribing. The individual cells of the matrix were populated with representative quotes that reflected intersecting domains to facilitate further analysis and aid with interpretation. Additionally, profiles were created for each participant using the codes within the broader theme *Beliefs about Consequences* due to its apparent significant as a behavioural determinant, in order to examine how varying beliefs influence opioid prescribing.

## Results

### Participants

A total of 22 FPs participated in a one-time interview. The average age was 41 years (range 31–61) and sex was relatively balanced between the two groups (refer to Table 1 for demographic details). The number of years in practice ranged between 2 and 32 years. The average practice size was 10 physicians, although two participants reported being the only physician in their practice.

**Table 1:** Participant Demographics

Participant characteristics		N (%)
Sex	Female	12 (55)
	Male	10 (45)
Practice type and funding model	Mainly capitation payment with government-funded allied health as part of the team	8 (36)
	Mainly capitation payment with no allied health	11 (50)
	Mainly fee-for-service with no allied health	3 (14)
Age [mean (range)]		41 (31–61)
Years in practice [mean (range)]		12 (2–32)
Years in current clinic [mean (range)]		10 (1–32)
Number of physicians in current clinic [mean (range)]		10 (0–74)

When analyzing the data according to demographic characteristics, more experienced physicians generally reported higher levels of confidence in prescribing opioids, which was attributed to strong therapeutic relationships. In the absence of specialized training (i.e., chronic pain management or addictions training), less experienced physicians tended to exhibit more conservative prescribing behaviours and adhered more closely to the opioid guideline recommendations. No additional patterns emerged when examining data according to the remaining demographic characteristics.

However we found that the nature of individual behavioral determinants and their interaction with one another explained the nuances underlying an individual physician’s opioid prescribing behavior. Relevant domains were not mutually exclusive; rather, the analysis revealed that their intersection created a 3 × 3 framework matrix (refer to Table 2). The TDF domain *Beliefs about Consequences* played a central role in explaining physician prescribing behaviours as they related to the management of chronic non-cancer pain. This theme intersected with emergent independent themes corresponding to the TDF domains *Beliefs about Capabilities*, *Behavioural Regulation*, and P*rofessional Role and Identity.* The TDF domains *Environmental Context and Resources* and *Emotion* also emerged as intersecting these three independent domains. An overview of quotes arranged by TDF domain can be found in the Additional file 2.

**Table 2:** 3 × 3 Matrix Outlining the Interaction between Behavioural Determinants of Opioid Prescribing

	**Beliefs about Capabilities** ***The physician’s belief of the truth or reality about their ability, talent, or facility that they can put to constructive use.***	**Behavioural Regulation** ***Anything aimed at managing or changing [the physician’s own] objectively observed or measured actions.***	**Professional Role & Identity** ***A coherent set of behaviours and displayed personal qualities of the physician in their work setting.***
**Beliefs about Consequences** ***The physician’s beliefs of the truth, reality, or validity about outcomes of their behaviour [or the behaviour of their patients] in a given situation.***	-Confidence in prescribing was influenced by individual beliefs about the risks and benefits of opioids -Limited evidence, the prevalence of chronic pain, and street supply leads FPs feeling that there is very little they can do	-Numerous unsuccessful experiences led to the belief that existing strategies were not sufficient to achieve guideline concordant care -Most FPs use a stepwise approach to pain management that aligns with guidelines, however this approach is grossly undermined by a lack of access or long waiting lists	-Tensions emerged between the FPs role as a “healer” who provides symptomatic relief and the need to avoid adverse consequences -Challenging conversations around opioid prescribing and pain management threaten the therapeutic relationship
**Environmental Context and Resources** ***Any circumstance of a physician’s situation or environment that discourages or encourages the development of skills and abilities, independence, social competence, and adaptive behaviour.***	-Poor access to mental health and addiction services and alternatives to pain management create a barrier to appropriately managing pain -Recent guidelines often had a neutral or negative influence on confidence in prescribing due to generally weak recommendations	-The system lacks effective resources to support FPs in monitoring opioid prescribing in their practice -Guidelines do not provide actionable suggestions for behaviours within the FPs immediate control (i.e., dose equivalent substitutions)	-Poor communication by specialists impedes the FPs ability to determine the appropriateness of extending certain prescriptions -The role of FPs vs. other prescribers in the system with respect to opioid prescribing and pain management are unclear, meaning that management often gets “dumped on” the FP
**Emotion** ***A complex reaction pattern, involving experiential, behavioural, and physiological elements, by which the physician attempts to deal with a personally significant matter or event.***	-Emotionally charged conversations with patients around pain management lead FPs to question whether they did the right thing -FPs do not feel equipped to navigate these conversations, creating a sense of anxiety in anticipation of these discussions -The perception of strong therapeutic relationships was perceived to diffuse emotional tensions and facilitate easier conversations	-FPs felt frustrated because there is minimal success in their strategies *-*Emotional consequences led some FPs to avoid prescribing as a mechanism to avoid these challenging conversations -There are currently no resources to help FPs diffuse the emotional tension that arises in challenging conversations	-Tensions around opioid prescribing and the need to police patients makes FPs feel terrible for not meeting their patients’ perceived needs -The FPs role as a “healer” is at odds with their role in provide guideline-concordant care, resulting in a range of conflicting emotions

*FP* family physician

### Beliefs about consequences

FPs narratives centred around the consequences stemming from prescribing opioids, specifically with respect to patient behavior and associated outcomes. A general sense of tension underscored the topic overall. Both prescribing and de-prescribing behaviours were associated with adverse patient outcomes and challenging encounters; however conflicting beliefs emerged across participants.

Tapering opioid prescriptions destabilizes patients and pushes them to the street

Consequences related to prescribing opioids were influenced by the perception of best practice guidelines, challenging encounters with patients, and previous experiences tapering high doses of opioids. FPs believed some aspects of newly released Canadian opioid guidelines encouraged actions that led to the destabilization of otherwise stable patients. Adverse consequences included the use of illicit drugs in response to tapering efforts and an increase in stimulant use.

*“In others, stimulant use and alcohol use goes way up when I titrate down their opioids. So, prescribing opioids in a controlled fashion for their pain, despite their pain risk, seems to be less risky.” P001*

The unintended consequence of using opioid contracts was highlighted, where patients became disconnected from the healthcare system, turned to illicit street use, and/or sought out a new prescriber who had no knowledge of their prior opioid use. FPs described a tension woven throughout their discussions with patients about their opioid use or misuse, creating tension between maintaining a trusting therapeutic relationship and adhering to the guidelines. These conversations were universally described as uncomfortable, leading to the anticipation of communication difficulties that FPs did not feel equipped to adequately handle.

*“Those conversations around ‘I don’t think prescribing this is appropriate’…physicians tend to shy away, because I think they expect them to be confrontational.” P003*

Most FPs felt that they lacked appropriate answers or solutions to assist patients in either buying-in or managing their symptoms during the tapering process. For some, being aware of the possibility of resistance allowed them to reflect and acknowledge the inherent difficulty in altering a therapy that may impact the patient’s functioning and daily activities. When attempts were made to decrease the dose of opioid to align with guideline recommendations, most physicians reported that pain symptoms were no longer managed, leading them to describe their tapering experiences as unsuccessful.

*“I think the challenge, for me, is when you talk about decreasing or trying to, patients kind of look at you and say, ‘But I still have pain. What do I do?’ And often, there are not many other available options.” P016*

*“Usually if it is successful it’s short-term and eventually [patients] come back and they say they can’t do it.” P017*

Conflicting views emerged with respect to the consequences of short versus longer acting opioids. For some, there was more comfort when prescribing short-acting opioids because physicians perceived a sense of control, or a “*leash*” on patients, while others believed short-acting opioids increased the likelihood of break-through pain. Long-acting opioids addressed the latter concern, however it increased the likelihood of a problematic situation whereby patients started down a path with addictive potential and escalating dosages.

Opioids have no role in my practice

A subset of FPs discredited the use of opioids in their non-cancer patient population citing limited indications and benefits, resulting in few, if any, opioid prescriptions generated by the provider themselves.

“*I, personally, other than cancer patients or palliative care patients, have never started anyone on chronic opioids and I never would. I see no role for it in my practice.*” *P020*

For those prescribing, a tension emerged between managing chronic pain and facilitating opioid misuse. FPs in this group held the belief that opioid prescribing was likely to create “*drug addicts*”, leading to the belief that opioid use conflicted with their professional identity in wanting to “*do the right thing*”. Patient misuse and abuse of opioids was frequently mentioned among these participants, however opinions differed as to what constitutes misuse. For example, some FPs reporting little use for opioids in their practice reported a misuse rate of around 5%, while others suggested misuse was occurring among 50 to 100% of their patients with an opioid prescription. One physician defined misuse as:

“…*using too much of it, period. And that, in itself, is a misuse in my mind. They become dependent on it and it becomes perpetuated and often accelerated. Don’t tell me that every now and then they don’t misuse, lose one, [and] come in a little bit early. If they come in early, then it’s a misuse*.” *P017*

Those who viewed misuse as a rare occurrence attributed increasing pain needs to aberrant behavior and gaps in the system. For example, a lack of timely access to primary care appointments was cited as a reason given for patients self-titrating their opioid medication. Several FPs described situations where patients were either ‘fired’ as a result of their misuse (as a violation of an opioid contract) or left the FPs care after an attempt at *“aggressive opioid tapering”*.

*“I got rid of those people. I stopped opioids on those people where it was a problem…or they left my practice and are probably getting it from another doctor. So, it’s hard to know if it’s successful. I said, no, you broke the opioid contract I had you on and here’s a tapering dose and that’s it. And then sometimes I just don’t see them again.” P002*

### Beliefs about capabilities

We’re trying our best but there’s not much we can do

Confidence in appropriately prescribing and managing patients on opioids was influenced by FPs perceptions of the consequences associated with opioid prescribing. Many expressed “*worry*” over the correct course of action when dealing with the possibility of their patients going into opioid withdrawal. Others who were more established in their practice (i.e., > 20 years of clinical experience) felt that they could manage opioid prescribing in a way that met the needs of their patients- a confidence that was largely attributed to their clinical experience and strong therapeutic relationships that reduced the perceived potential of adverse consequences. Previous experiences, such as working in the shelter system or an addiction facility, and opioid-specific training also bolstered confidence.

*“I have a bread and butter family medicine practice, cradle to grave. I probably prescribe about two patients a week for acute pain, a limited prescription, and then I probably have about 30 to 35 patients who are on chronic opioids. Acute, it’s not really a concern. I know my patients, I have a steady practice. So if I have a time limited prescription for a purpose that a person’s pulled their back post-surgery, dental, you know, they’ll get 10 to 20 and then never again, I’m not concerned about that.” P012*

Identifying the underlying cause of pain was highlighted as a challenge. FPs emphasized that available evidence only supported opioid prescribing for a select few diagnoses, which could often be determined objectively (e.g., via imaging). In contrast, FPs cited a range of emotional and psychosocial components contributing to chronic non-cancer pain, complicating their ability to establish a clear diagnosis. These contributing factors also influenced the ability to manage patients currently receiving opioid therapy, as these patients often pushed back when the appropriateness of the current dose was questioned.

*“The challenge would be the patient that almost tries to pressure you into it because they say ‘I can’t live without it… my pain is so chronic’… you try to wean them down, and they’re really insistent.” P011*

“O*nce you get patients on them, it’s almost impossible to get them off. They just sort of latch onto them.*” *P015*

The availability of street drugs was highlighted as a significant concern that influenced the prescribing patterns of FPs. Participants felt that it was safer for high-risk patients to be accessing opioids through the healthcare system under the care of a consistent provider instead of turning to illicit drug use. The availability of such drugs was unanimously acknowledged as beyond the control of FPs, however its impact was acutely tied to the perceived degree of control that FPs have over managing the overall opioid crisis.

“*There are certainly a lot of people who started off just needing opioids for chronic, non-cancer pain and end up in clinics for opioid agonist therapy because they have a severe opioid use disorder. Then they escalate into street opioids. So, that’s a big problem*” *P001*

System resources don’t support pain management

The perceived inability to appropriately manage pain was influenced by the lack of accessible, non-pharmacological options within the Ontario healthcare system. Long wait lists coupled with cumbersome referral processes for pain, addiction, and substance abuse services often discouraged physicians from pursuing these treatment options, or required them to find an appropriate pain management strategy in the interim period. Several FPs indicated that although guidelines recommend a multi-disciplinary approach, community resources are either unavailable or financially inaccessible for many patients. A small sample of rural FPs recognized the absence of patient services and pursued additional training in opioid prescribing and management “*out of necessity*”.

“W*here’s the support? Yeah, but where’s the multi-disciplinary approach? There aren’t any community resources out there to help us.*” *P018*

*“It takes some time to find a resource, for physio or for massage, all those other things that could help manage pain. And it takes some time to get those in place. There’s not another great alternative pain-management mechanism out there, both pharmacological and even again, non-pharmacological. Often, [patients don’t qualify], and they can’t get access to a lot of the other supports.” P016*

The recent guidelines were often cited as a neutral or negative influence on confidence due to the overall lack of strong evidence, undermining their utility. *“Weak recommendations”* led FPs to question the efficacy of suggested alternatives to opioid prescribing, resulting in frustration and the sentiment that prescribing habits were being *“challenged”*. FPs often relied on consultation with colleagues, including other FPs, pharmacists, and occasionally pain specialists, to determine their course of action for a given patient.

*“As a primary care physician, you’re being told to treat pain and to acknowledge patients’ pain and to do something about it. And so, it’s very difficult to walk that line. And all of those guidelines start with medications that are largely ineffective, for most people’s pain.*” *P008*

The contextual features of an individual FP’s practice also influenced perceived confidence in their abilities. “*Knowing the patient*” strengthens confidence, owing largely to familiarity with the patient’s mental health history, addiction risk, and the overall potential of the opioid for optimizing function. Patient beliefs around opioid use also impacted FP confidence, with much higher confidence levels noted when considering prescribing to patients who express concern or avoidant behaviour with respect to starting opioid therapy. For those physicians who worked in an interdisciplinary environment, the social influence of their colleagues improved their confidence in their prescribing practices and management strategies.

Uncertainty and anticipation causes stress

Emotional undercurrents influenced FP’s confidence in their ability to prescribe according to guidelines. Most participants described feeling a sense of dread, frustration, or a strong dislike when engaging in discussions with patients around opioid use. FPs described ongoing worries once the encounter was over, highlighting the uncertainty surrounding prescribing decisions. The ambiguity that surrounds prescribing decisions was also a source of stress among many participants.

“*It can be a tough call sometimes. When I do prescribe I’m a little bit uneasy.*” *P003*

*“Whenever there’s some ambiguity and you’re not sure about something, that causes stress.” P004*

The emotional consequences of prescribing led to some physicians wanting to avoid opioid prescribing altogether. This was compounded by the anticipation of challenging conversations that many FPs felt ill equipped to navigate effectively, occasionally resulting in a sense of fear. Consequently, these physicians were likely to shy away from or altogether avoid having to confront their patients about opioid misuse. The length of the therapeutic relationship was described as a determinant of prescribing self-efficacy, as it relates to communication, as a trusting relationship was often viewed as diffusing emotions and enabling easier conversations around tapering or opioid avoidance.

“*I think the ones who trust me, knowing that I’m trying to help, won’t leave angry.*” *P001*

### Behavioural regulation

Current strategies are not able to overcome barriers

FPs employed a variety of strategies to help regulate and monitor opioid prescribing in their practice, including the use of opioid contracts, random urine drug screens, and liaising with local pharmacists to inquire about patient prescribing history. Gradual tapering was a common method, however most participants felt they had little control over tapering long-standing opioid prescriptions, owing largely to previously failed attempts coupled with patients’ reluctance to change.

“*And if they’re very stabilized, try to taper them, but so far, everyone I taper comes back a month later really upset and in pain, and I have to put them back up.” P001*

Many FPs identified sources of leverage to justify tapering to their patients; these strategies included telling patients that they could “*lose [their] license*” for inappropriately prescribing opioids, or that they “*have to follow*” the guidelines. Some physicians used avoidance rather than tapering as a method of regulating opioid misuse, due to the uncertainty surrounding indications and the perceived risks of chronic opioid use.

*“I don’t even bring it up often as an option, just because I can see the issues that people have once they’re on it, that we can’t get them off opioids.” P019*

FPs described their clinical decision-making process, which involved weighing the risks and benefits of opioid use as they would for any other prescription. This decision-making process was reflective of the FPs need to find the “*right balance*” while addressing patients’ pain management needs.

*“The bottom line is, if someone is in such pain that I think that they would benefit from opioids, and there is no other modality or treatment that I think would be helpful in that circumstance, then I prescribe opioids. I weigh the risks and the benefits of opioids much more than I do with almost any other prescription that I write.” P008*

Most physicians used a step-wise approach to pain management when dealing with new presentations of pain. This involved the optimization of non-pharmacological therapies prior to opioid prescription; however, the utility of this approach was largely undermined by the reality that non-pharmacological therapies were often inaccessible or ineffective.

The guidelines summarize evidence, but don’t provide enough guidance

There was a clear lack of resources available to help FPs self-monitor opioid prescribing in their practice. The recent guidelines were the most prominent external resource, however the recommendations were noted to be “*largely ineffective, for most people’s pain*”. Several physicians indicated that the guidelines offered no form of “*guidance*”, as there was a notable absence of self-monitoring tools and strategies; recommendations provided general suggestions instead of clear actions; significant barriers existed with respect to non-pharmacological strategies; and the guidelines were not easily applied to complex cases.

We’re all frustrated and no one is happy

FPs were frustrated by persisting challenges despite their attempts to employ a range of strategies within their practice. Previous experiences with patients expressing “*resistance*” or “*anger*” led to the anticipation of conflict and a visceral reaction of fear when patients requested opioids during their appointment. These emotional consequences led some FPs to avoid opioid prescribing altogether as a mechanism to avoid challenging conversations.

“Y*ou become on guard right away. Whether rightfully or wrongfully, maybe to some extent, rightfully. But you right away think, okay, it’s not just like my average, easy visit. This is going to be something that I’ve got to be more challenging and more aware.*” *P016*

The lack of available resources amplified the stress experienced by FPs, with many FPs not “know[ing] what else to offer” when examining options for pain management. The skills to successfully navigate challenging conversations with patients consistently eluded FPs in the absence of guidance or training to promote effective communication strategies.

*“There are the ones that will put up resistance, and almost have a sort of anger toward the prescriber for suggesting tapering, and not saying yes. Then that becomes stressful because there’s a lot of friction.*” *P013*

### Professional role and identity

Every doctor wants to do the right thing

FPs described their perspectives on opioid prescribing against the backdrop of their professional identity as a healer, where their goal is to address the symptoms and decrease the suffering their patients experience. A key tension emerged between the need to “*treat and acknowledge patients’ pain and do something about it*” and “*being told that you’re prescribing too much and you’re doing harm*”. While some felt that the negative attention was unfair, others acknowledged the role that physicians have played in contributing to the opioid crisis. These conflicting identities further complicated decisions around whether to prescribe.

“*I think it’s a very difficult balance, because there’s certainly a lot of harm done by opioid prescribing by physicians. Physicians are at least responsible for controlling the supply of prescription opioids.*” *P001*

Challenging conversations around opioid prescribing and pain management were believed to threaten the therapeutic relationship, undermining physicians’ professional identity. Many FPs struggled to balance their identity as a healer and as a physician who must not worsen the prevalence of addiction, as both identities were associated with adverse consequences.

*“I think every doctor wants to do the right thing. I think 99.9%, unless they’re selling prescriptions or whatever. I think most doctors need more to do the right thing, because we didn’t go into this profession to create drug addicts.” P018*

It always gets dumped on the family physician

Role conflict was partially attributed to the organization of the healthcare system, specifically poor communication between prescribers (i.e., specialists and FPs) and poor access to alternative treatment options to address chronic pain and addiction. FPs detailed case examples where opioids were initially prescribed by a surgeon or specialist, who instructed the patient to follow-up with their family doctor. The prevailing belief is that specialists rarely do their due diligence by communicating the treatment regimen and rationale to FPs, leaving them unable to judge the appropriateness of continued prescribing when requested by the patient. Coordinating services and completing cumbersome referrals magnified the challenge of managing misuse and addiction- responsibilities for which the FPs perceived fell to them as the result of an inefficient system.

“*It always goes back to the family doctor. It’s very rare the specialist is going to say, well, now I’ll take over this, I’ll prescribe their opioids. It’s more often that they’re like, here’s some advice, go follow up with your family doctor. So, it really does circle back to the family doctor the majority of the time and [they’re] left with figuring out how to decrease that opioid prescription or stop it altogether.*” *P002*

Competing priorities and the time-constrained nature of family practice created a barrier to the successful management of chronic pain, particularly with respect to patient education. “*Maintaining a good standard of care*” was articulated as the primary goal, with many FPs indicating the need for more active supports to support improvements in care.

“*And I think that is tough in our busy practices, to actually take time to really educate people about pain, and that’s why we offer this chronic pain program through our family health team…But you can imagine a physician that doesn’t have access to that. You don’t generally have the time to spend in sessions talking about how to manage pain.*” *P004*

Not serving the patient makes you feel terrible

The significance of maintaining a trusting and positive therapeutic relationship was the central source of emotion amidst an emerging trend suggesting physicians need to “*police themselves*”.

“*Yeah, it’s very difficult because if I have a doubt about them, it’s very hard for a physician to say no to pain management. It’s very hard, you feel like you are not serving the patient properly and the good con artists will make you feel like you are a piece of you know what… So, it’s very hard to say no when we’re told that one of our jobs is to relieve pain and suffering.*” *P017*

Balancing the need to “*relieve pain and suffering*” while attempting to diagnose based on the patients’ subjective expressions of pain was described as negatively impacting the therapeutic relationship. Some FPs described the anger elicited during these conversations, resulting in the physician “*yelling*” at patients when they try to reason for a higher dose. Prescribing was further complicated by the FPs role in public accountability and upholding medical standards. This professional role resulted in an overall “*fear*” of prescribing for many FPs due to the potential of “*repercussions*” from their regulatory body.

“*You want your patients to trust you, to believe you, you want the clinical encounter to be relatively conflict free. I mean, I think that’s the biggest struggle, and I think some doctors, as a result, don’t prescribe opioids. Which is wrong, because they’re a legitimate clinical and pharmacological resource, but there’s that fear.*” *P012*

## Discussion

This study qualitatively explored FPs perceptions of opioid prescribing with the objective of understanding perceived barriers and enablers to guideline-adherent management of chronic non-cancer pain. Individual beliefs about the consequences of prescribing opioids and subsequent opioid (mis)use by patients were a central determinant across all narratives. Emotions arising in relation to opioid prescribing and the perceived lack of access to helpful resources (environmental context) also played a key role. Each of these three key domains interacted with the prescriber’s beliefs about their capability to safely manage opioid therapy, their behavioural regulation strategies, and their perceived professional role and identity. The interaction between these behavioural determinants help explain variation in opioid prescribing behaviours and suggest that interventions to improve the safety of opioid prescribing will need to be both tailored and multifaceted.

Nearly a decade ago FP’s self-reported opioid management revealed substantial knowledge and practice gaps that were not in line with recent guidelines [9]. The knowledge gap appears to have diminished but FPs continue to experience challenges translating their knowledge to practice, underscoring the need for training in the area of CNCP management and addictions across the profession [29]. Participants reported variable use of opioid guidelines, noting a lack of congruence between recommendations and level of access to non-pharmacological therapies or evidence-based multidisciplinary support within the system; unfortunately, publicly funded treatment for these interventions remains scarce in Canada [30]. This finding, considered alongside sustained prescribing variation, suggests that opioid prescribing guidelines alone are insufficient to influence opioid-prescribing behaviour and the management of CNCP [31]. Between 40 and 45% of previously surveyed physicians with opioid prescribing experience reported lengthy wait lists for pain specialists and treatment facilities, and inadequate consultations and referral resources for chronic pain management [8, 32]. Furthermore, chronic use of prescription opioids in the non-cancer patient population is more common in patients with mental health and substance use disorders [33–35]. Optimal treatment for individuals with comorbid disorders has been demonstrated in care management models and collaborative care pain interventions [36, 37]. However, most patients cannot access to the evidence-based multidisciplinary treatment approaches recommended in the guidelines and prescribing practices are impacted by these contextual variables [38]. Thus, interventions that connect patients in need with the supports that may benefit them seem to be warranted.

FP participants expressed frustration at dealing with the burden of managing opioids in a system where they perceive specialist physicians to be the primary initiators of these prescriptions. Poor communication between providers regarding follow-up plans results in FPs feeling uncertain about whether to renew or taper opioids [39]. Over 86% of Ontario FPs report high confidence in opioid prescribing, which is positively associated with the number of patients prescribed opioids [40]. However, this confidence likely reflects increasing comfort with commonly prescribed medications rather than confidence adjusting prescribing patterns to reflect guidelines. This suggests a need for interventions that develop capacity amongst health professionals to communicate effectively about opioids – both with patients and with each other.

Strongly held patient expectations for pain medication prescriptions further challenge FP’s ability to appropriately prescribe as they strive to provide patient-centred care. Emotionally-charged interactions occur in response to disagreement over opioid-related action plans, which in turn negatively affects doctor-patient communication and collaboration [41]. Ongoing prescribing of an opioid becomes an easier option that allows prescribers to avoid uncomfortable encounters [42]. The management of CNCP relies heavily on patient-provider communication and interactions can become contentious [43, 44], requiring FPs to rely on their communication skills to navigate conversations. Effective communication strategies include validating pain experiences, being honest about not having all the answers, reviewing previous treatments before beginning conversations about treatment options, providing follow-up instructions, clearly explaining how patients may benefit from referrals, and sharing personal experiences of chronic pain when applicable [45]. Engaging in shared decision making is likely to yield short-term improvements in patient confidence and satisfaction in addition to having a more distal impact on the patient-provider relationship through establishing a norm where collaboration and deliberation become expected behaviors [46]. Strategies include providing the patient with individualized rationale for tapering, reassuring the patient that they will not be abandoned during the tapering process, and providing opportunities for collaboration [43]. Providing patients with treatment options is a well-received approach to negotiating treatment and can include offering the option to taper dose versus switch to another opioid or cutting back the number of pills versus reducing the dose of each pill [43]. Patient-facing solutions (i.e., decision aids) have been utilized to decrease postoperative opioid prescribing and include information on (1) anticipated patterns of pain; (2) expected use; (3) risks and benefits of opioid and non-opioid analgesics; and (4) opioid disposal and access to refills (if needed) [47].

The goal of this qualitative study was not to generalize but rather to provide a rich, contextualized understanding of FPs perspectives on opioid prescribing by way of an in-depth study of particular cases. Future work should explore whether these findings are consistent across the broader population of FPs in Ontario and Canada. Participants in the current study self-reported low rates of opioid prescribing in their practice, therefore findings may not be reflective of those who exhibit above average prescribing rates. The accuracy of physician self-report is limited [48], therefore future work exploring determinants of prescribing behavior would benefit from triangulation with quantitative performance data to objectively explore whether and how behavioral determinants influence variations in performance. Despite a broad recruitment strategy, the study sample is not geographically diverse. The majority of participants were from the Greater Toronto Area, with a few participants residing in northern and eastern Ontario. Our sample size to reach saturation was similar to other studies that have used TDF to focus on specific prescribing behaviours [49–51]. We acknowledge that using TDF domains as pre-defined codes may have obscured the emergence of other codes or ways of interpreting the data, and that TDF domains and constructs can be interpreted differently [52]. Applying the TDF provided a well-developed theoretical underpinning and a common language, enabling the identification of evidence-based behavioural determinants and the ability to compare with previous studies.

## Conclusion

FPs face a wide range of complex (and often interacting) challenges when prescribing opioid therapy to their patients in a climate of increased prescriber scrutiny. To our knowledge, the interactions between determinant domains described in this study provides novel insights into the real-world determinants of FPs prescribing behaviours. Solution-based strategies to support improved prescribing and management of opioids should target these determinants directly using evidence-based strategies that move beyond guideline dissemination and general education. Shared decision-making strategies, including providing the patient with individualized rationale for tapering, reassuring the patient that they will not be abandoned during the tapering process, and providing opportunities for collaboration is likely to reduce tension.

## Additional files

Additional file 1: Study interview guide. Questions that guided semi-structured interviews with participants. (PDF 84 kb)

Additional file 2: Overview of theoretical domains framework and exemplary quotes. Definitions of relevant TDF domains and exemplar quotes from across participant interviews. (PDF 83 kb)

## Abbreviations

ED: Emergency department
FFS: Fee for service
FP: Family physician
HQO: Health Quality Ontario
TDF: Theoretical Domains Framework
