# Supplementary material for: Understanding the behavioural determinants of opioid prescribing among family physicians: a qualitative study
Source: BMC Fam Pract. 2019 May 10;20:59. doi: 10.1186/s12875-019-0947-2 (PMC6511163; doi:10.1186/s12875-019-0947-2)
Supplement: Supplementary file 1 — Study interview guide. Questions that guided semi-structured interviews with participants. (PDF 84 kb) [file 12875_2019_947_MOESM1_ESM.pdf]

Appendix: Study Interview Guide

1. Tell me a bit about your practice and the patients receiving opioid pain medication.

2. How often is incorrect or inappropriate use of opioids a problem among your patients.

3. What do you think are some of the challenges family physicians are facing right now in terms of prescribing opioids and managing chronic pain?

   Follow-up: Which of these challenges have you experienced in your practice?

4. Can you describe the factors beyond your control that influence opioid prescribing?

5. Are you aware of any resources/guidelines to help you prescribe opioids for chronic non-cancer pain? If so, which ones?

   Follow-up: To what extent do these tools/clinical guidelines/professional recommendations influence your opioid prescribing?

6. How do you decide whom to prescribe opioids for?

   Follow-up: Are certain patients better candidates than others? Any that are not good candidates for opioids?

7. How do you decide what actions to take when a patient complains about chronic pain and has no previous history of opioid use?

   Follow-up: What factors help you recognize chronic pain in your patients?

8. How do your considerations differ when potentially prescribing an opioid for the first time (e.g., new start) versus reviewing an existing prescription?

9. How comfortable are you in prescribing opioids to your patients?

   Follow-up: What might make you feel more comfortable?

10. Do you consult with colleagues in patient cases that involve opioid use if you have a question or concern?

11. Overall, is opioid prescribing difficult or frustrating to deal with?
    Follow-up: **If YES**, why do you feel that way?

12. Do you see a need for supportive interventions to improve opioid prescribing? Why or why not?

13. What type(s) of direct support for primary care might be helpful?

Follow-up: How would this support help improve opioid prescribing or the management of patients with acute or chronic pain?

14. What barriers would these supports be addressing in your practice?

15. How do you envision this support might fit within your regular work routine?

16. Are there any incentives that could be put in place to encourage primary care physicians to adhere to opioid prescribing guidelines?

17. How can primary care physicians like yourself be motivated to adhere to opioid prescribing guidelines?

Thank you. Do you have any additional thoughts or comments that we haven't yet discussed?
