# Supplementary material for: Understanding the behavioural determinants of opioid prescribing among family physicians: a qualitative study
Source: BMC Fam Pract. 2019 May 10;20:59. doi: 10.1186/s12875-019-0947-2 (PMC6511163; doi:10.1186/s12875-019-0947-2)
Supplement: Supplementary file 2 — Overview of theoretical domains framework and exemplary quotes. Definitions of relevant TDF domains and exemplar quotes from across participant interviews. (PDF 83 kb) [file 12875_2019_947_MOESM2_ESM.pdf]

Supplementary Table 1- Relevant TDF Domains

Theme domain	Definition	Sample quotes
1. Beliefs about consequences	Acceptance of the truth, reality, or validity about outcomes of a behaviour in a given situation	*"In [some patients], stimulant use and alcohol use goes way up when I titrate down their opioids. So, prescribing opioids in a controlled fashion for their pain, despite their pain risk, seems to be less risky… like I know the patient injects once in a while, sometimes tapering them off can lead to these adverse outcomes, so I'm just still trying to find the right balance.*" P001<br><br>*"There are a few that when you get to the much lower dose there probably are issues around their physical dependence on the medication, but more so probably the mental dependence. So, they actually feel that if they go off it means they won't be able to cope properly. The pain is going to rebound quite significantly, so initially when you're tapering opioids, when you get to those lower doses you do start to have these challenges."* P003<br><br>*"[Some] people aren't actually using the opiates that they're being given or, they're using something else like cocaine and then are fired and cut off cold turkey, so the classic case of unintended consequences. So we have to be careful about that aspect of education but many patients sell and trade their opiates for other things, just money or other drugs."* P022
2. Beliefs about capabilities	Acceptance of the truth, reality about an ability, talent, or facility that a person can put to constructive use	*"I developed advanced addiction, opioid, and mental health scales out of necessity, not necessarily out of interest. I applied and have had a Methadone exemption for pain, I'm going to say 15 years now."* P014<br><br>*"[If a patient is] coming to me with [chronic pain] already established, then I'm sort of trying to chase after the previous medical records and see just how legitimate it is, that kind of thing."* P015
3. Behavioural regulation	Anything aimed at managing or changing objectively observed or measured actions	*"I'm pretty militant in my prescribing patterns. So, they get only a certain number of weeks at a time. I do regular urine drug opioid screening as well. They don't get any early renewals unless there's like a good documented reason."* P002

		"I try to use anti-inflammatories whenever I can, but sometimes because of intolerance or comorbidities, I can't use it 100%. Then that's when I usually will start the short-acting opioids, and then sometimes you progress to a long-acting when it becomes chronic." P013
4. Professional role & identity	A coherent set of behaviours and displayed personal qualities of an individual in a social or work setting	*"I'm there to lead my patients and treat them according to what they tell me. I can't function under the guise of suspicion."* P012<br><br>*"We're often stuck as a family doctor to give them the benefit of every doubt and we're often releasing [prescriptions] early, giving them an extra one every month or two or five every month."* P017
5. Environmental context & resources	Any circumstance of a person's situation or environment that discourages or encourages the development of skills and abilities, independence, social competence, and adaptive behaviour	*"If I felt outside my comfort zone, you know, the default right or wrong would be not to prescribe and refer to a pain clinic. For example, I don't have the ability to have a supervised urine test. I don't have a nurse and our bathrooms are outside our office. It's quite frustrating with these guidelines talking about doing drug testing and whatnot. It's just not feasible in a regular family medicine office."* P012<br><br>*"My patients with chronic opioids, a majority of them, not majority, all of them came to me already on these medications prescribed by other doctors. The community and I took over their care and continued their prescriptions, fortunately or unfortunately."* P020
6. Emotion	A complex reaction pattern, involving experiential, behavioural, and physiological elements, by which the individual attempts to deal with a personally significant matter or event	*I think on an individual basis, it can be challenging to confront a patient who's kind of entrenched in taking opioids and try to have that conversation about whether you're tapering it fully and stopping or tapering it to some degree. The conversations can also be quite difficult when you're faced with some kind of inconsistency in how they're taking it and you can raise that with them. So, I think from a kind of individual physician point of view, it can be pretty stressful to manage that and keep track of."* P002

		"There are [patients] that will put up resistance, and almost have a sort of anger toward the prescriber for suggesting tapering, and not saying yes. Then that becomes stressful because there's a lot of friction." P013
